# Senolytics improve bone forming potential of bone marrow mesenchymal stem cells from aged mice

**DOI:** 10.1038/s41536-021-00145-z

**Published:** 2021-06-11

**Authors:** Yueying Zhou, Xiaonan Xin, Lichao Wang, Binsheng Wang, Li Chen, Ousheng Liu, David W. Rowe, Ming Xu

**Affiliations:** 1 Hunan Key Laboratory of Oral Health Research & Hunan 3D Printing Engineering Research Center of Oral Care & Hunan Clinical Research Center of Oral Major Diseases and Oral Health & Xiangya Stomatological Hospital & Xiangya School of Stomatology, Central South University, Changsha, Hunan China; 2 UConn Center on Aging, Farmington, CT USA; 3 Center for Regenerative Medicine and Skeletal Development, Farmington, CT USA; 4 Department of Genetics and Genome Sciences, UConn Health, Farmington, CT USA

**Keywords:** Senescence, Mesenchymal stem cells

## Abstract

The osteogenic potential of bone marrow mesenchymal stem cells (BMSCs) declines dramatically with aging. By using a calvarial defect model, we showed that a senolytic cocktail (dasatinib+quercetin; D + Q) improved osteogenic capacity of aged BMSC both in vitro and in vivo. The study presented a model to assess strategies to improve bone-forming potential on aged BMSCs. D + Q might hold promise for improving BMSC function in aged populations.

Bone marrow mesenchymal stem cells (BMSCs) are a population of multipotent progenitor cells that have regenerative potential of various tissues^1^. With aging, the function of BMSCs declines dramatically^1,2^, and limited interventions exist to rejuvenate BMSC population from aged donors.

The underlying mechanisms of age-related changes in BMSCs are not fully characterized. With aging, a portion of BMSCs might become senescent like other MSCs^3^, and contribute to these changes. Cellular senescence refers to the stable arrest of cellular proliferation^4^, and might impact aged BMSCs function by both intrinsic and extrinsic mechanisms. Recently, various senolytic drugs have been developed to specifically kill senescent cells. The cocktail of dasatinib and quercetin (D + Q) is the first reported senolytic agents, and they eliminate senescent cells by transiently suppressing senescence-associated anti-apoptotic pathways which are highly activated in senescent cells and protect these cells from surrounding pro-apoptotic microenvironment^5^. D + Q has been shown by us and others to reduce senescent cell burden and improve tissue function in various tissues with aging including fat^6^, bone^7^, aorta^8^, and brain^9^. Notably, systemic administration of D + Q in aged mice demonstrates an increase in bone volume and suppression of osteoclast activity^7^. However, it remains unknown whether D + Q has any direct impact on osteogenic potential of aged BMSCs.

In this study, an in vitro cell culture and in vivo transplantation model was used to assess the effect of D + Q on inherent osteogenic potential of BMSCs derived from old donors. BMSCs cultures were established from young (3 month-old) and old (27 month-old) male mice, and were treated with vehicle (V) or D + Q (0.2 µM+20 µM) for 24 h^5^. At the end of the 24 h treatment period, an ATP-based assay revealed that D + Q-treated old BMSC cultures had 20–30% lower cell number than V-treated cells while the reduction of cell number in young BMSCs by D + Q was only 10% (Fig. 1a). Similar findings were observed when actual cell numbers were counted (Supplementary Fig. 1a). In addition, more apoptotic cells were observed in D + Q treated BMSCs (Supplementary Fig. 1b, c). These results indicate that D + Q has age-preferential killing effects on certain cell populations in BMSCs. The cultures were stained with senescence associated beta-galactosidase (SABG), a classic biomarker for cellular senescence in vitro. Old BMSCs contained more SABG + cells (20%) than young BMSCs (10%) and D + Q reduced SABG + cells in old (10%) but not young BMSCs (Fig. 1b,c). Additionally, D + Q reduced the expression levels of several senescence-related and inflammation markers including p21, p16, Il6, Cxcl1 and Mcp1 in old BMSCs (Fig. 1d), similar to other aged tissues^6,7^. Notably, this effect was conserved between BMSCs from old male and female donors (Supplementary Fig. 2a).

**Fig. 1:**
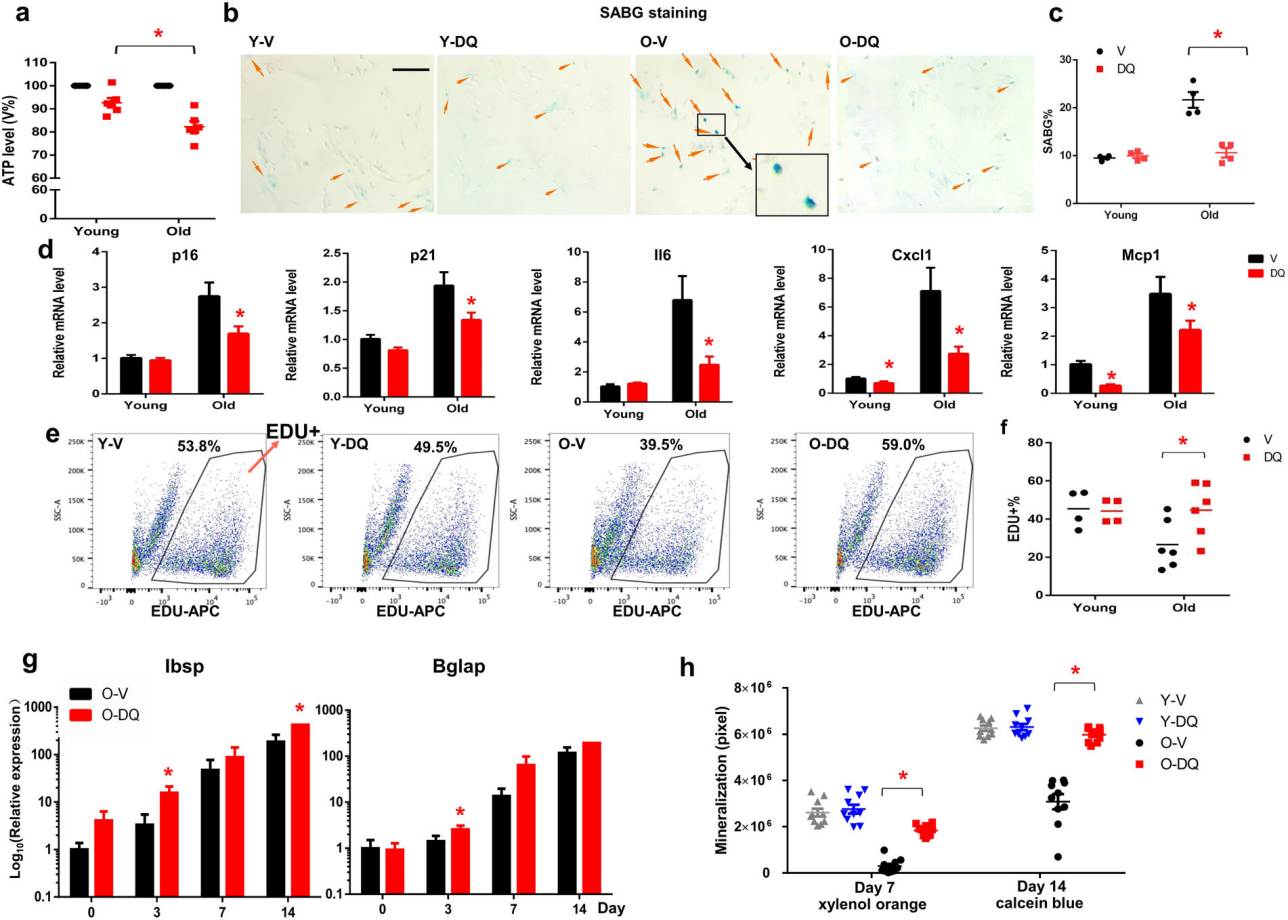
D + Q improved proliferation and osteogenic capacity of old BMSCs in vitro. **a** ATP assay of young and Old V or DQ treated BMSCs. *n* = 6 for both groups. **b** Representative images of senescence-associated β-gal (SABG) staining. Scale bar: 50 μm (**c**) Percentage of SABG positive cells. *n* = 4 for both groups. **d** Expression of p16, p21, Il6, Cxcl1, MCP1 was analyzed by real-time PCR at day 0. *n* = 10 (male and female donors combined) for both groups. **e** FACS analysis on EDU + cells. **f** Percentage of EDU + cells of the total cells. *n* = 4 for young groups and *n* = 6 for old groups. **g** Expression of Ibsp and Bglap was analyzed by real-time PCR at day 0, 3, 7, and 14 after the exposure to osteogenic media. *n* = 6 for both groups. **h** Old V or DQ treated BMSCs were differentiated in osteogenic media. The same wells were stained with xylenol orange at day 7 and calcein blue at day 14 to quantify the mineralization area at both time points. *n* = 10 (male and female donors combined) for both groups. Results were shown as means ± s.e.m. *p < 0.05; two-tailed Student’s t-test.

After 24 h recovery period in the culture media without D + Q, the BMSCs were tested for proliferation and osteogenic differentiation. The proliferation rate was assessed using the incorporation of 5-ethynyl-2′-deoxyuridine (EdU) during the S-phase of cell mitosis. Using fluorescence-activated cell sorting (FACS), the old BMSCs had a lower proliferation rate (30%) than young BMSCs (45%), and D + Q significantly increased the proliferation rate in old (40%) but not in young BMSCs (Fig. 1e, f). We next determined if D + Q could improve the in vitro osteogenic capacity of old BMSCs. Ibsp (bone sialoprotein) and Bglap (osteocalcin) are two key players for osteogenesis, and are widely used osteogenic markers. The expression levels of these two genes were higher in D + Q treated old BMSCs at several time points after exposure to osteogenic media (Fig. 1g). Concordantly, using mineral staining on the whole well, D + Q treated old BMSCs had more xylenol orange staining on day 7 and increased calcein blue staining on day 14 compared to V-treated cells. D + Q had little effect on differentiation capacity of young BMSCs (Fig. 1h). Importantly, the effect of D + Q on osteogenic differentiation potential was similar between BMSCs from male and female donors (Supplementary Fig. 2b, c). These results suggest that D + Q could reduce senescence, increase proliferation and improve osteogenic differentiation of old BMSCs in vitro.

The effect of D + Q on the bone forming potential of old BMSCs in vivo was assessed using the calvarial defect model. D + Q- or V-treated old BMSCs as well as young BMSC cultures (5×10^5^ cells/defect) were loaded into collagen-hydroxyapatite (HA) scaffolds and implanted into calvarial defects in 3-month-old immuno-deficient NOD.Cg-Prkdc^scid^-Il2rg^tm1wj1^/SzJ (NSG) mice which carry a Col3.6GFPtpz transgene^10^. Implanted BMSCs will generate a bone organoid composed of a cortical like bone shell with a well-developed marrow space^11^. This structure is dependent on the remodeling of the initially deposited bone matrix that is driven by the host osteoclast and the ability of the donor cells to continue to generate functional osteoblasts. Calvarial were harvested at 6 weeks after a single i.p. injection of alizarine complexone (AC) 1 day prior to sacrifice to identify active mineralizing bone surfaces. X-rays of the dissected calvarial revealed that the area of newly formed mineralized tissue relative to the defect size (Fig. 2a,e) was significantly lower in calvaria transplanted with V-treated old BMSCs (50%) compared to both young (90%) and D + Q-treated old cells (80%). Frontal histological sections of the dissected calvaria revealed that the V-treated old BMSCs failed to develop a similar sized marrow space as the young or D + Q group (Fig. 2b, f). Interestingly, the contribution of GFP-expressing host cells in the implant tissue was greater in the V-treated old cells than DQ ones (Fig. 2c, g). The calvarial defects transplanted with V-treated old cells had lower TRAP (tartrate-resistant alkaline phosphatase, a marker for osteoclast activity) signal (Fig. 2d, j) on the endocortical surface and outer cortical shell compared to other groups, indicating reduced bone resorption. AP (alkaline phosphatase, an enzymatic indicator of osteoblast activity) signals relative to the cortical bone area were similar among 3 groups (Fig. 2d, h) while the total AP signals were lower in V-treated old BMSCs (Fig. 2i). AP reflects the layer of differentiated osteoblasts on the bone surface and these findings suggest that the coupled remodeling activity between osteoblasts and osteoclasts was impaired in Old-V group, and the reduced osteoclast but continued osteoblast activity results in the smaller marrow space and widened cortical shell. In summary, D + Q treatment improved the osteogenic capacity of old BMSCs, and resulted in bone organoids with restored bone remodeling and an enlarged and functional bone marrow space.

**Fig. 2:**
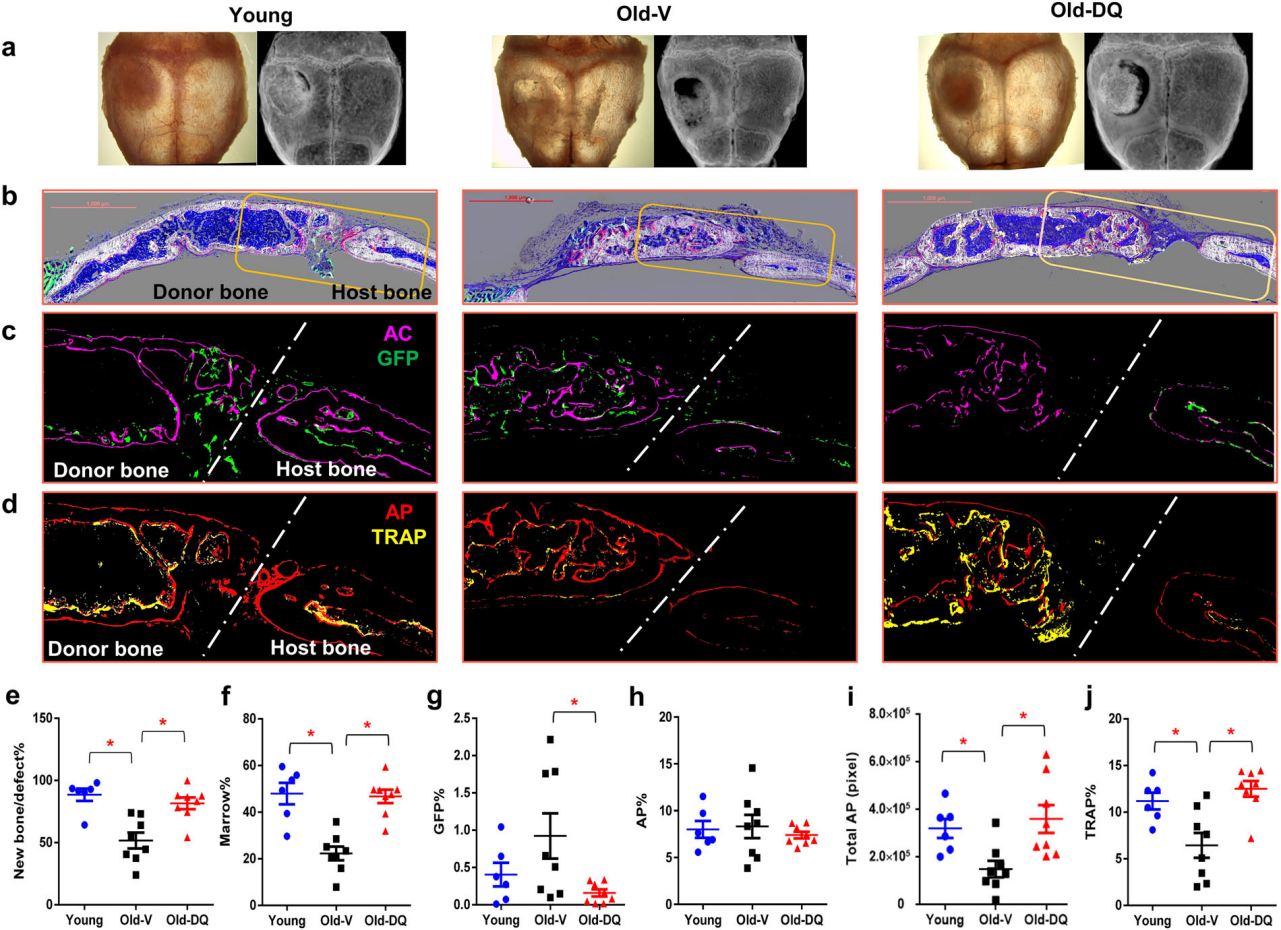
D + Q improved osteogenic capacity of old BMSCs in vivo. **a** Representative pictures and X-ray images of the whole calvarial defects. **b** Representative multimodal histological images of calvarial defects with mineral (white), AP (red), TRAP (yellow), GFP (green), AC (purple), TB (dark blue) and DAPI (blue) staining. Scale bar: 1000 μm. Enlarged images of calvarial defects with (**c**) AC + GFP signal and (**d**) AP + TRAP staining. **e** New Bone Area as percentage of the Defect Area. **f** Marrow area as percentage of the total cortical shell area. **g** GFP area as percentage of the total cortical bone area. **h** AP linear surface area as percentage of the total cortical bone area. **i** Total AP linear surface area. **j** TRAP linear surface area as percentage of the total cortical bone area. Results were shown as means ± s.e.m. *n* = 6 for Young, *n* = 8 for Old-V, *n* = 8 for Old-DQ. *p < 0.05; two-tailed, unpaired Student’s t-test.

Although D + Q has been shown to alleviate various age-related tissue dysfunction, it is still unclear whether D + Q could improve old BMSC function. Our study, for the first time, demonstrated that D + Q could improve osteogenic capacity of BMSCs isolated from aged mice. Notably, the effects of D + Q on BMSC senescence and osteogenic potential were similar between two sexes, consistent with previous findings on other bone cells^7,12^. In addition to more mineralized tissue formation demonstrated by X-rays and in vitro studies, histology analysis of the implant model showed 3 major benefits of DQ treatment that are not revealed by in vitro models: (1) more bone marrow space. The ability of BMSCs to create an environment for marrow development and subsequent bone remodeling is a key attribute for judging the competency of osteogenic cells^13^; (2) more TRAP activity, indicating increased bone remodeling and osteoclast contribution; (3) less contribution from host cells. Unless stimulated by osteogenic growth factors^14^, the host calvarial-derived mesenchymal cells rarely contribute to new bone formation when a competent donor population is implanted into the defect space. Thus, this indicates that the function of donor BMSCs is likely to be improved. It is also possible that the V-treated old BMSCs are impeding the remodeling activity of the host cells that migrated into the organoid, which needs to be tested by future studies. Importantly, all these findings in D + Q group were similar to the young group, suggesting that D + Q might rejuvenate “old BMSC population” to a youthful condition.

Mechanistically, we postulate that clearance of senescent cells is one of the major mechanisms by D + Q. In spite of relatively low (~10–20%) percentage in old BMSCs, senescent BMSCs have little osteogenic potential (autonomous) and could greatly impair osteogenic capacity of non-senescent BMSCs (non-autonomous), likely through SASP and resulting inflammation^15^. Notably, both D and Q have a wide range of biological functions in addition to senolytic property. Due to the facts that we only treated old BMSCs with D + Q for 1 day, the long-lasting effects in vitro and in vivo suggest that the mechanisms are more likely to be cell population change (such as senescent cell elimination) than the ones requiring continuous presence of the drugs, such as inhibition/activation of enzymes or pathways. Moreover, D + Q improved osteogenic capacity only in old BMSCs, but not in young BMSCs. All these findings suggest that improved osteogenesis of old BMSCs by D + Q is at least partially due to clearance of senescent cells. Cell population changes by aging and D + Q could be another potential mechanism, which needs to be investigated using single cell sequencing in future. In addition, future studies are needed to further explore the role of senescent cells using transgenic mouse models, potential sex difference in vivo, the effects of D + Q on young cells in vivo, and other underlying mechanisms of impaired regenerative potential in old BMSCs.

The calvarial defect model and the associated cryohistological evaluation of the non-decalcified repair tissue^16^ is an informative and efficient platform to assess the osteogenic potential of BMSCs in vivo. It reveals properties of bone progenitor cells that cannot be appreciated by in vitro studies. Unlike treating aged mice with D + Q, this model enabled us to distinguish the effect of donor BMSCs from non-autonomous effects caused by the aged host, because the host mice were 3-month-old with minimal impairment on osteogenesis capacity. Moreover, this model has a rapid turnaround time. We were able to examine the effect of D + Q on old BMSCs within 2 months, which is suitable and efficient for potential drug screening. In future, this model can be leveraged to screen and validate various interventions targeting aging mechanisms of old BMSCs to improve their regenerative potential. This model can also be used to test the role of other senescent cell types such as osteocytes on bone regeneration.

In summary, our study provides a model for simultaneously evaluating the cellular basis for the in vitro and in vivo effects of senolytic agents on the old BMSCs. D + Q does appear to be beneficial for restoring the bone and bone marrow forming potential of old BMSCs. These agents along with other senolytic compounds hold promise for improving BMSC function in aged populations.

## Methods

### Material and mouse models

All animal experiments were performed according to protocols approved by the Institutional Animal Care and Use Committee (IACUC) at UConn Health. Wild-type C57BL/6 mice were obtained from the National Institute on Aging (NIA) and maintained in a pathogen-free facility at 23–24 °C under a 12-h light, 12-h dark regimen with free access to a standard mouse diet (Teklad global 18% protein, Envigo #2918, Indianapolis, IN) and water. Dasatinib (LC Laboratories, Woburn, MA) and quercetin (Sigma-Aldrich, St Louis, MO) were dissolved in DMSO for in vitro treatment. Scaffold discs (3.5 mm diameter × 0.5 mm thickness) were purchased from Healos HA (DePuy Orthopaedics, Raynham, MA). Alexa Fluor647 dye, ELF^®^97 phosphatase substrate, and cryomatrix™ were purchased from Thermo Fisher Scientific (TWaltham, MA, USA). Fast Red-TR and Naphthol AS-MX Phosphate was obtained from Sigma-Aldrich. All other materials were purchased from Thermo Fisher Scientific.

### BMSC preparation

BMSCs were isolated from 3-month and 27-month (old) C57BL/6 mice. The epiphyseal growth plates of the femurs and tibias were removed, and the bone marrow was collected by flushing with α-minimum essential medium (α-MEM). The total marrow suspensions were prepared by gently mixing the cells with a pipette. Cells were centrifuged at 6000r/min for 1 min and plated at a density 3 × 10^6^/cm^2^ on 100 mm dish containing 10 mL cell culture medium that consists of α-MEM with 10% fetal bovine serum and 1% penicillin-streptomycin. At day 3 of seeding, complete medium was replaced with fresh α-MEM culture medium and maintained in culture until the cultures became 80% confluent after which it was passaged to two 100 mm dishes. When the passaged cell reached 80% confluency, the cultures were treated with 0.2 µM dasatinib+20 µM quercetin or vehicle (0.1% DMSO) for 24 h, washed, and allowed to recover for 1 additional day. The dose of DQ was chosen based on a previous publication to kill more senescent cells without killing many non-senescent control cells^5^. These BMSCs (passage 1) were used for both in vivo implantation and in vitro characterization.

### Calvarial defect model

A 3.5 mm defect in the left parietal bone was introduced in 3-month-old immune-deficient NSG mice carrying a Col3.6GFPtpz reporter transgene. NSG mice were anesthetized with ketamine (135 mg/kg)/xylazine (15 mg/kg). The head was shaved and the surgical site was cleaned with 75% ethanol. An incision was made just off the sagittal midline to expose the parietal bone. The pericranium was removed, and 3.5 mm defects were made on left sides of nonsuture-associated parietal bone using a trephine drill. V or D + Q treated old BMSCs as well as young BMSCs were harvested and 5 × 10^5^ cells/defect were absorbed into HA scaffolds discs, and implanted into the defect space. Prior to sacrifice at 6 weeks, mice were injected intraperitoneally with alizarin complexone (AC; 30 mg/kg body weight) one day before the sacrifice.

### Histology tissue preparation and staining

All the procedures were described previously^10^. Calvaria were dissected from the skull, fixed in 10% formalin, and imaged radiographically at 4× magnification (6 s at 26 kVp) using a digital capture X-ray cabinet (Faxitron LX-60). Quantitative new bone formation was calculated by dividing the new bone formation size by the total defect size. The calvaria were then transferred into a 30% sucrose solution in PBS after 4 days of formalin fixation. The tissues were positioned in Shandon cryomatrix^TM^. Cryosections (6μm) through the nondecalcified calvaria were obtained on a Leica CM3050S cryostat (Leica, Wetzlar) using a disposable steel blade (Thermo Scientific). For bright field imaging, sections were initially imaged under differential interference contrast (DIC) optics using AxioScanZ1 slide scanner (Carl Zeiss). Endogenous fluorescence of Col3.6Tpz was detected with Yellow Fluorescent Protein (YFP) (yellow-green) filter. AC labeling was detected with MCherry filter. This combination of YFP, MCherry, and DIC enabled visualization of the endogenous fluorescence of the Tpz (recipient) mice while highlighting the newly formed mineralized layer in the nondecalcified section. The GFP signal represent for contribution from host mice in new bone formation is examined by counting the number of green pixels divided the total number of pixels in the regeneration mineral area. The section was then stained for TRAP for which the slides were covered with freshly prepared preincubation solution (112 mM sodium acetate, 76 mM sodium tartrate, and 11 mM sodium nitrite, pH 4.1-4.3) at room temperature for 10 min. The slide was then drained and covered with the same buffer now containing 60 μM ELF^®^97 phosphatase substrate followed by UV exposure for about 5 min at room temperature. The reaction was stopped by submerging the slides with three changes of PBS for 10 min with gentle agitation. Slides were cover slipped with 50% glycerin in PBS and imaged with a combination of yellow filter optimized for tetracycline (Chroma Technology Custom HQ409sp, 425dcxr, HQ555/30, set lot C-104285) and YFP filter. Next, the slides were incubated in the AP reaction buffer (100 mM Tris, pH 9.5, 50 mM MgCl2, 100 mM NaCl) for 10 min for AP staining followed by incubation in reaction buffer containing 0.2 mg/mL Fast Red TR and 0.1 mg/m Naphthol AS-MX Phosphate for 5 min. After washing in PBS, the section was covered with 50% glycerol containing 10 μg/mL of Hoechst 33342 and the slide was cover slipped for imaging using a combination of Tomato (red) and 4′,6-diamidino-2-phenylindole (DAPI) filters. This step was followed by toluidine blue (Tol Blue) counterstaining to view nuclei/tissue matrix by bright field imaging. We examined TRAP and AP activity by counting the number of yellow and red pixels within linear surface area divided by the total number of pixels in the regeneration mineral area. The marrow percentage is marrow area divided by the total area of newly formed bone.

### ATP assay

After V or DQ treatment, cell number of young and old BMSCs were tested using Luminescent ATP assay (CellTiter-Glo^®^2.0 Cell Viability, Promega) as previously described^3^.

### SABG Assay

Cellular SABG activity was assayed as previously described^17^. In brief, cells were fixed for 5 min in PBS containing 2% (vol/vol) formaldehyde (Sigma-Aldrich) and 0.25% glutaraldehyde (Sigma-Aldrich). Following fixation, cells were washed three times with PBS before being incubated in SABG activity solution containing 1 mg/ml 5-bromo-4-chloro-3-indolyl-β-D-galactoside (X-Gal) in 0.1 M citric acid buffer (pH 6.0) at 37 °C for 14-16 h. The enzymatic reaction was stopped by washing cells with cold PBS for 3 times. About 8–10 images were taken using fluorescence microscopy (Nikon Eclipse Ti) from random fields in each sample. DAPI was used to stain nuclei for cell counting.

### Apoptosis assay

FITC Annexin V Apoptosis Detection Kit with PI (BioLegend, #640914, San Diego, CA) was employed according to the manufacturer’s instruction. Cells were analyzed by BD LSR II flow cytometer (BD Biosciences, San Jose, CA).

### EdU proliferation assay

EdU (10 μM) were added into culture media 14-16 h before collection. Then, cells were washed three times with PBS before harvest by 0.25% trypsin-EDTA. Cells were fixed in 4% paraformaldehyde for 15 min in dark, and permeabilized by 0.3% Triton X-100. Then these cells were incubated in EdU staining buffer containing 2 mM CuSO4, 10 mM Ascorbic Acid, 100 mM Tris buffer (PH 7.4) and 5 μM AF647 Fluorescent Azide for 30 min in dark. After wash, cells were re-suspended in media containing 2% FBS for FACS analysis.

### RNA extraction and quantitative real-time PCR analysis

Total RNA was extracted using TRIzol reagent (Thermo Fisher), purified with chloroform, isopropanol and 75% ice cold ethanol (Sigma-Aldrich), dissolved in RNase free water, and then reverse transcribed to cDNA with M-MLV reverse transcriptase kit (Thermo Fisher). Quantitative real-time PCR (qPCR) was conducted using PerfeCTa^®^ FastMix^®^ II (Quantabio,

Beverly, MA) on CFX96 Real-Time PCR detection system (Bio-Rad Laboratories, Hercules, CA) previously described^18^. Taqman probes and primers were purchased from Integrated DNA Technologies (IDT, Coralville, Iowa). The internal control housekeeping gene used is TBP (TATA-binding protein) and TBP levels were not significantly different between all groups. Primer sequence information is included in the Supplementary Information.

### Osteogenic differentiation

When cell density reached to 80%, osteogenic media containing 2 μL/mL ascorbic acid and 4 μL/mL β-glycerophosphate were added to BMSCs. BMSCs were harvested at day 0, 3, 7, 14 for RNA extraction. For mineral staining, xylenol orange (20 μM) was added to osteogenic media at day 6 after differentiation for 12 h. Cells were washed, and imaged by fluorescent microscope using a TRITC Red filter (red signal). Calcein blue (30 μM) was added to osteogenic media at day 13 after differentiation for 24 h in the same well, and cells were imaged using a DAPI filter (blue signal).

### Reporting summary

Further information on research design is available in the Nature Research Reporting Summary linked to this article.

## Supplementary information

Supplementary Information

Reporting Summary

## Data Availability

The data that support the findings of this study are available from the corresponding author upon request.
