# Supplementary material for: Senolytics improve bone forming potential of bone marrow mesenchymal stem cells from aged mice
Source: NPJ Regen Med. 2021 Jun 11;6:34. doi: 10.1038/s41536-021-00145-z (PMC8195980; doi:10.1038/s41536-021-00145-z)
Supplement: Supplementary file 1 — Supplementary Information [file 41536_2021_145_MOESM1_ESM.pdf]

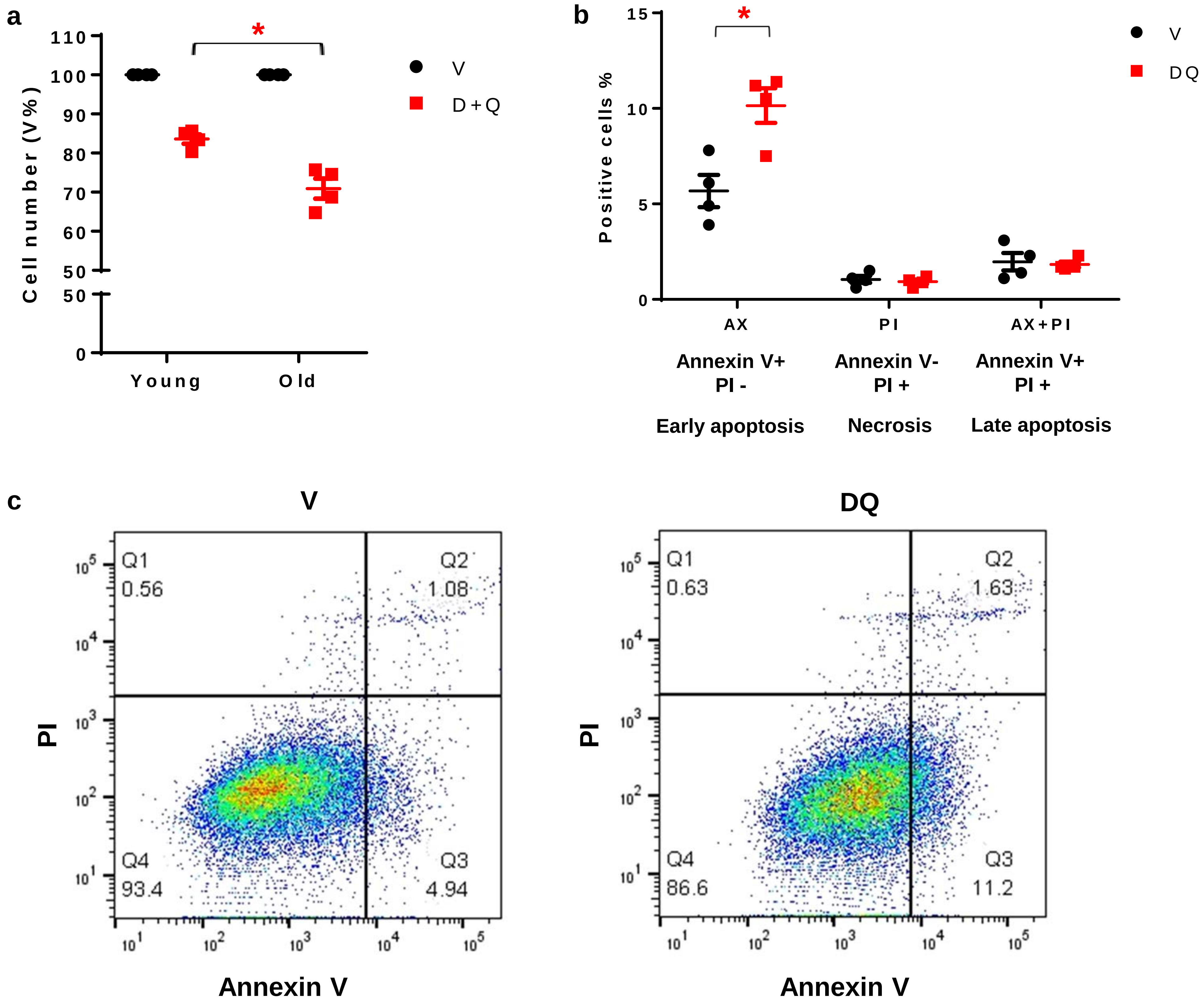

Supplementary Figure 1

Supplementary Figure 1. D+Q killed senescent cells through apoptosis. (a) Cell counts of young and Old V or DQ treated BMSCs. n=4 for both groups. (b) Percentage of Annexin V positive, PI positive and double positive cells. n=4 for both groups. (c) Representative images of FACS analysis on Annexin V and PI staining. Results were shown as means ± s.e.m. *, $p < 0.05$; two-tailed Student's t-test.

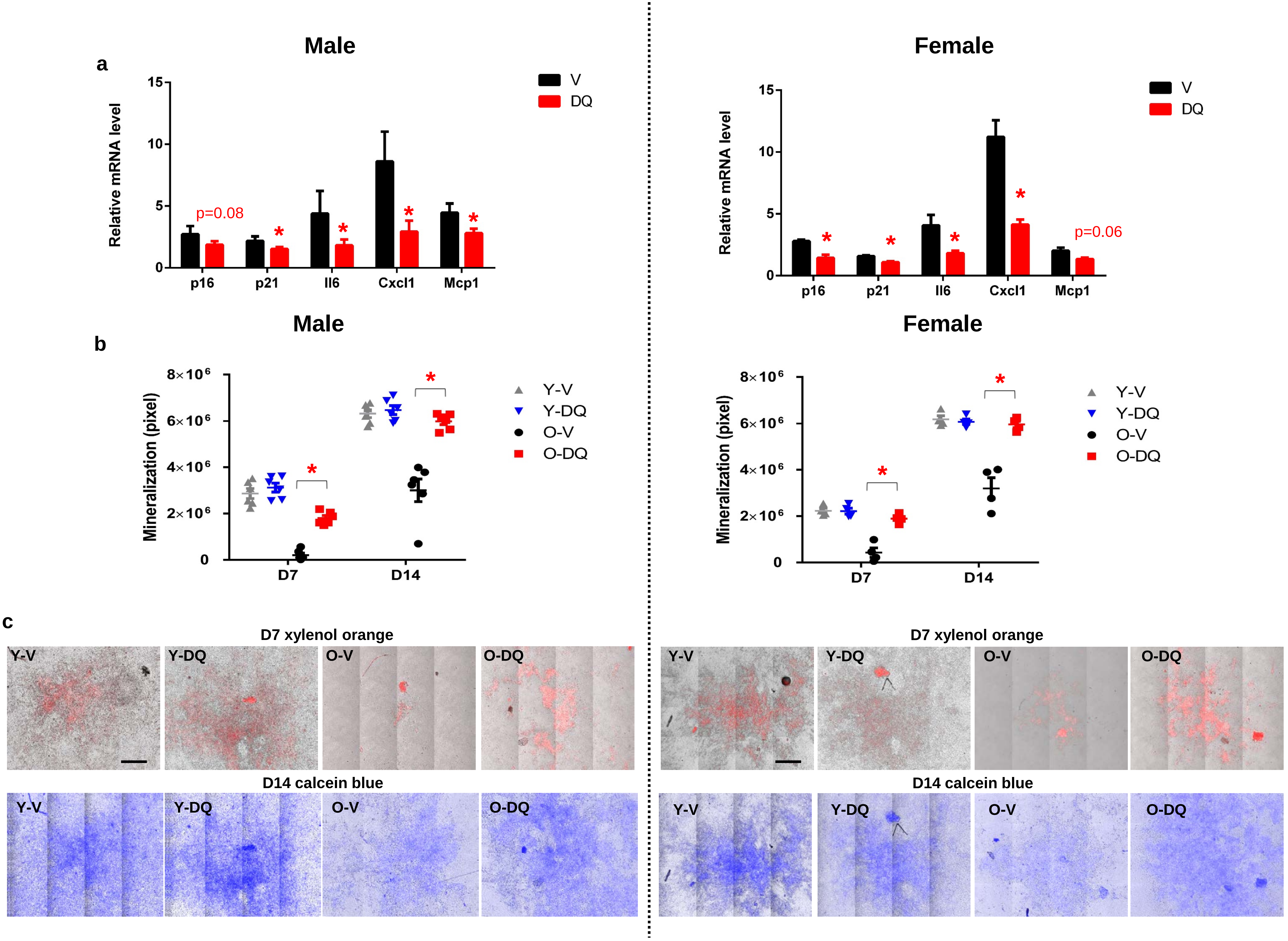

Supplementary Figure 2

Supplementary Figure 2. D+Q reduced senescence and improved osteogenic capacity of old BMSCs from both male and female donors. (a) Expression of p16, p21, Il6, Cxcl1, MCP1 was analyzed by real-time PCR at day 0 for BMSCs from old male and female mice. n=6 for male and n=4 for female donors. (b) Old V or DQ treated BMSCs were differentiated in osteogenic media. The same wells were stained with xylenol orange at day 7 and calcein blue at day 14 to quantify the mineralization area at both time points. n=6 for male and n=4 for female donors. (c) Representative images of xylenol orange staining and calcein blue staining. Results were shown as means ± s.e.m. *, $p < 0.05$; two-tailed Student's t-test. Scale bar: 500um

Primer sequence information

Gene	Catalog Number		Sequence
TBP	Mm.PT.39a.22214839	Probe	/56-FAM/ACT TGA CCT /ZEN/AAA GAC CAT TGC ACT TCG T/3IABkFQ/
		Primer-1	TGT ATC TAC CGT GAA TCT TGG C
		Primer-2	CCA GAA CTG AAA ATC AAC GCA G
IL6	Mm.PT.58.10005566	Probe	/56-FAM/CCT ACC CCA /ZEN/ATT TCC AAT GCT CTC CT/3IABkFQ/
		Primer-1	AGC CAG AGT CCT TCA GAG A
		Primer-2	TCC TTA GCC ACT CCT TCT GT
p16	Mm.PT.58.8388138	Probe	/56-FAM/CGC ACC GGA /ZEN/ATC CTG GAC CA/3IABkFQ/
		Primer-1	TGT TGT TGA GGC TAG AGA GGA
		Primer-2	AAT CTG CAC CGT AGT TGA GC
p21	Mm.PT.58.17125846	Probe	/56-FAM/TTC AGA GCC /ZEN/ACA GGC ACC ATG T/3IABkFQ/
		Primer-1	CAG ATC CAC AGC GAT ATC CAG
		Primer-2	GAA GAG ACA ACG GCA CAC T
Cxcl1	Mm.PT.58.42076891	Probe	/56-FAM/AGG TGT CCC /ZEN/CAA GTA ACG GAG AAA GA/3IABkFQ/
		Primer-1	CCA AAC CGA AGT CAT AGC CA
		Primer-2	GTG CCA TCA GAG CAG TCT
Mcp1	Mm.PT.58.42151692	Probe	/56-FAM/ACT CAC CTG /ZEN/CTG CTA CTC ATT CAC C/3IABkFQ/
		Primer-1	CAT CCA CGT GTT GGC TCA
		Primer-2	AAC TAC AGC TTC TTT GGG ACA
Bglap	Mm.PT.58.9119501.g	Probe	CCCAGACCTAGCAGACACCATGAG
		Primer-1	AGCAGAGTGAGCAGAAAGATG
		Primer-2	GAACAGACAAGTCCCACACAG
Ibsp	Mm.PT.58.29341883	Probe	ATGAAGACCAGGAGGCGGAGG
		Primer-1	GAGAGTGTGGAAAGTGTGGAG
		Primer-2	AGAAAATGGAGACGGCGATAG
